# Assessment of Veterinary Antimicrobial Residues in Feedlot Soils in Argentina

**DOI:** 10.3390/antibiotics15090862

**Published:** 2026-09-04

**Authors:** Sergio Sánchez Bruni, Laura Galotta, María Guadalupe de Yaniz, Laureano Schofs, Mónica Sparo, Lucila Cantón, Laura Moreno, Peers Davies

**Affiliations:** 1 Faculty of Veterinary Sciences, Universidad Nacional del Centro de la Provincia de Buenos Aires (UNCPBA), Tandil B7000, Buenos Aires, Argentina; gdeyaniz@vet.unicen.edu.ar (M.G.d.Y.); lschofs@vet.unicen.edu.ar (L.S.); lmoreno@vet.unicen.edu.ar (L.M.); 2 Tandil-Veterinary Research Center (CIVETAN), CONICET-UNCPBA-CICPBA, Tandil B7000, Buenos Aires, Argentina; msparo@salud.unicen.edu.ar (M.S.); cantonlu@vet.unicen.edu.ar (L.C.); 3 Independent Researcher, Arrecifes B2748, Buenos Aires, Argentina; galottalaura@vet.unicen.edu.ar; 4 Faculty of Health Sciences, Institute of Research in Health Sciences, Universidad Nacional del Centro de la Provincia de Buenos Aires (UNCPBA), Olavarría B7400, Buenos Aires, Argentina; 5 Department of Livestock & One Health, Institute of Infection, Veterinary and Ecological Sciences, University of Livepool, Leahurst Campus, Neston CH64 7TE, Cheshire, UK; peers.davies@liverpool.ac.uk

**Keywords:** antimicrobials, feedlots, environment, soil, residues, One Health

## Abstract

**Background/Objectives**: This prospective cross-sectional study quantified veterinary antimicrobial residues in soils from 27 commercial beef feedlots across multiple regions of Argentina and production scales. **Methods**: Using a validated Ultra-Fast Liquid Chromatography–tandem mass spectrometry (UFLC-MS/MS) method, we measured 15 target antimicrobials in pooled corral soil samples. **Results**: Monensin was detected in all feedlots and showed the highest detection frequency and widest concentration range; peak concentrations exceeded 15,000 ppb in some sites. Oxytetracycline presented pronounced, site-specific peaks (approaching 14,000 ppb), consistent with its strong adsorption to soil organic matter and limited biodegradation. These results indicate localised hotspots of antimicrobial loading in feedlot environments. These elevated concentrations signify substantial antimicrobial loading in confined animal environments and potential hotspots for environmental selection pressure. Compounds absent from farm treatment records were detected in 40.7% of feedlots, correlating with antimicrobial residues not matching available AMU records, including oxytetracycline, tulathromycin, cefquinome, enrofloxacin, and ciprofloxacin among others. Multiple non-exclusive explanations are plausible (historical persistence, manure redistribution, incomplete records, off-label use, or cross-contamination). The detection of critically important antimicrobials (e.g., fluoroquinolones and advanced cephalosporins) in soils raises One Health concerns about environmental selection pressure and the potential dissemination of resistance determinants. **Conclusions**: These findings underscore gaps in stewardship and the need for integrated environmental monitoring and improved antimicrobial usage traceability. These data provide a baseline for ecological risk assessment and resistome studies and can inform evidence-based policy and stewardship interventions in Argentine feedlot systems.

## 1. Introduction

**Figure 1:**
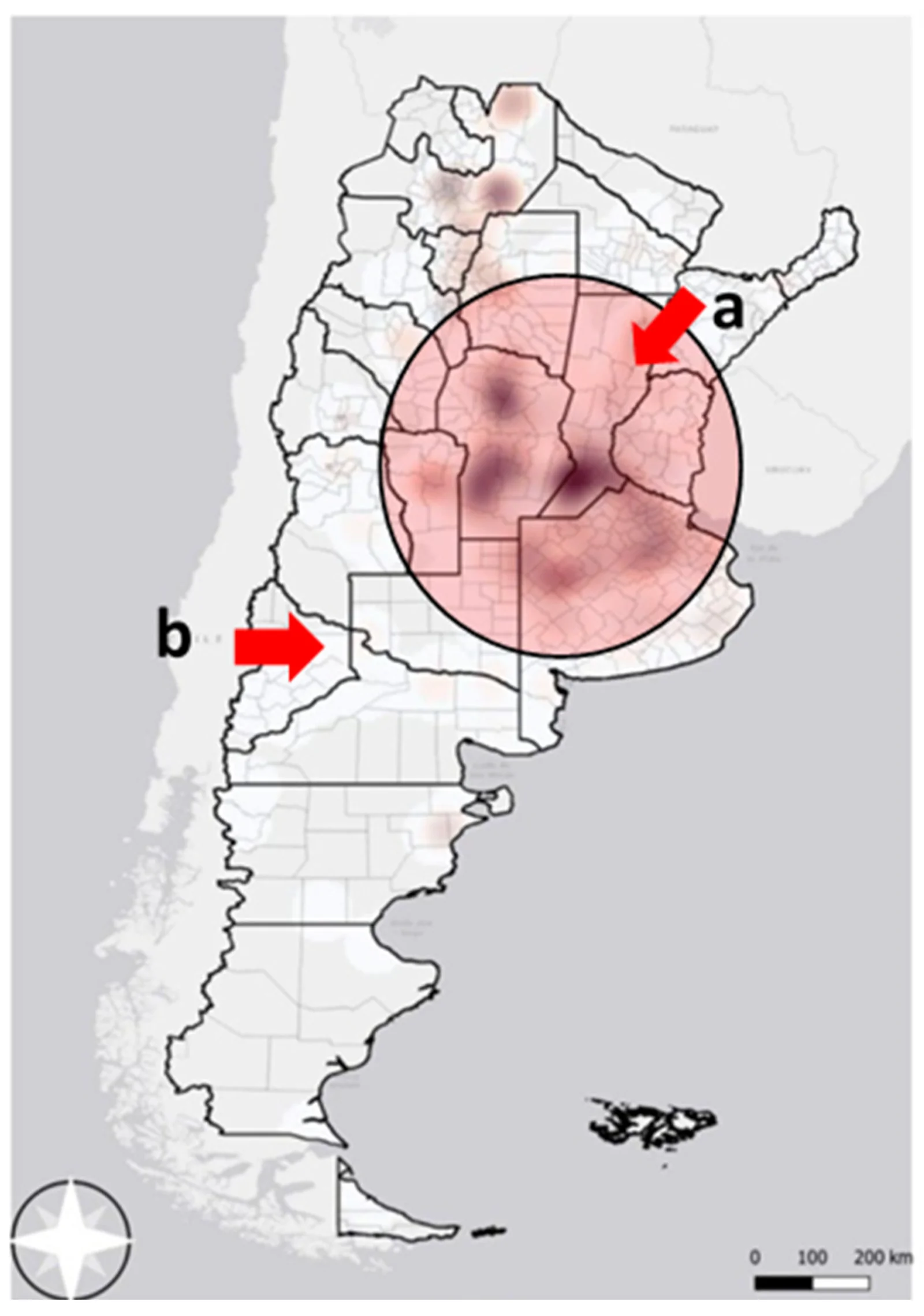
Distribution of recruited feedlots farms: (a) Central area (*n* = 21), 10,000 over 40,000 heads/farm, (b) Patagonia (*n* = 6), <500 heads/farm.

As livestock production has intensified, there has been a steady rise in the use of antimicrobials (ATM) in beef cattle feedlots. This use sits at the crossroads of animal, environmental, and public health, which is why it is a major focus in the One Health approach [1]. When antibiotics are given to food animals, a significant portion is excreted into the environment through manure, contaminating the soil and water runoff. This creates a pathway for antibiotic residues to spread, which not only contaminates the ecosystem but also represents a selection pressure for antimicrobial resistance (AMR) in a wide range of bacterial species in the wider ecosystem [2,3,4]. Whenever antimicrobials are given to animals—whether to prevent (prophylaxis), control (metaphylaxis), or treat disease (therapeutic)—a significant amount passes through their bodies unchanged or as active metabolites. These drug residues accumulate in the soil when manure is spread [5,6]. In feedlots, where animals are managed at high stocking densities and antibiotic treatments are frequent, this can create hotspots of contamination. Soil is not a passive bystander; it holds onto antibiotics, where they stick to organic matter, break down only partially, or influence soil microbial population dynamics. Even low levels of antibiotics in the soil can encourage bacteria to exchange resistance genes, adding to the challenge of antimicrobial resistance [7,8]. The persistence and mobility of antibiotics in soil depend on their physicochemical properties, including solubility, pKa, and adsorption coefficients, as well as environmental variables such as pH, texture, temperature, and organic carbon content [6,9]. Tetracyclines, sulfonamides, macrolides, and β-lactams are among the most frequently used classes in beef production worldwide and are commonly detected in agricultural soils impacted by animal manure [4,6]. Previous studies conducted in North America, Europe, and Asia have reported detectable concentrations of veterinary antimicrobials in soils adjacent to intensive livestock operations [5,10]. Despite growing global evidence, data from South American beef-producing systems remain limited. Argentina ranks among the world’s leading beef producers, with feedlot finishing systems expanding considerably over the past decades. However, systematic environmental surveillance of antibiotic residues in Argentine feedlot soils is scarce. Existing regional research has predominantly focused on antimicrobial resistance phenotypes or water contamination [11]. However, quantitative assessments of soil residues remain largely unexplored. This lack of baseline data limits risk assessment and restricts the implementation of integrated One Health monitoring frameworks. Argentina has a total of 50,000,000 heads of cattle (meat and dairy) distributed mainly in the centre and the northeast of the country. The strategic regions of the feedlots recruited for this study are illustrated in Figure 1.

Beef cattle production is 3 million tons/year, coming from a total of 12,100,979 heads of cattle, with circa 2,000,000 heads subjected to the feedlot system. Feedlots of cattle in Argentina vary from 1000 to 90,000 heads according to the farm size.

The present prospective cross-sectional study aimed to evaluate the occurrence and concentration of veterinary antimicrobial residues in soils from commercial feedlots in Argentina. A total of 27 feedlots were included, representing different regions and operational scales. Soil samples were analysed using validated Ultra-Fast Liquid Chromatography coupled with tandem mass spectrometry (UFLC-MS/MS) multi-ATM residue methods. We hypothesised that measurable concentrations of antimicrobial residues would be detected in a substantial proportion of evaluated feedlots, reflecting antimicrobial usage patterns in intensive beef production. By generating primary data from a major beef-producing country, this trial contributes to closing a significant regional knowledge gap and supports evidence-based environmental risk assessment within the One Health framework.

## 2. Results

### 2.1. Antimicrobial Use (AMU): Reported Antimicrobials

**Figure 2:**
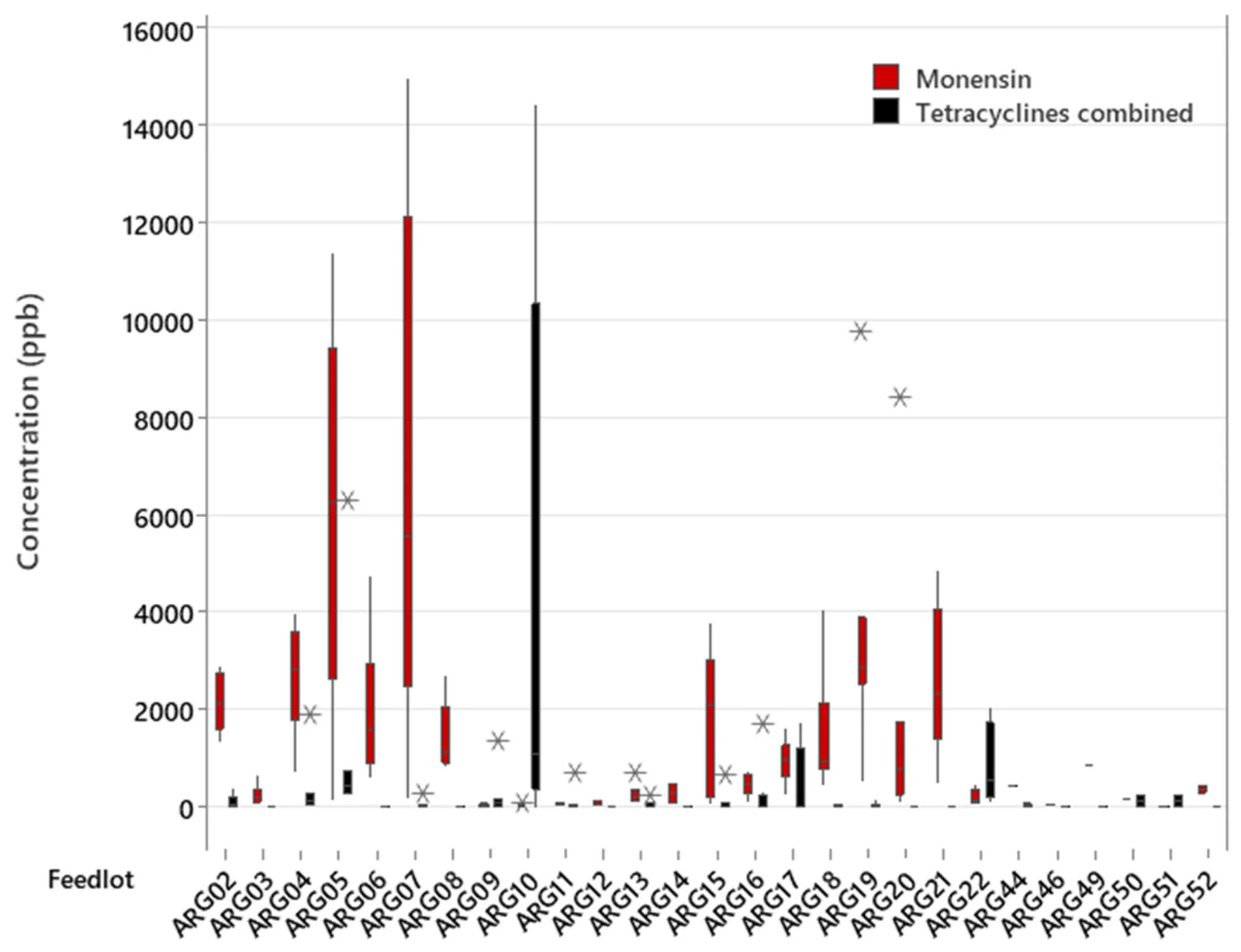
Distribution of residue concentrations of monensin and tetracycline antibiotics (tetracycline combined-oxytetracycline and chlortetracycline) in soil samples from corrals in each farm. Outlier values for individual corrals are denoted by an asterisk.

Monensin (MON) residues were detected in samples from all feedlots. Residues of other antimicrobial classes (most commonly tetracyclines) were detected in 70% (19 of 27) of the evaluated feedlots, with marked variability in both detection frequency and concentration among compounds and sites (Figure 2) and with antibiotic residues of up to five different classes detected in individual corrals.

MON exhibited the highest overall detection frequency and the broadest concentration range, with multiple feedlots showing elevated levels. Peak concentrations were observed in ARG05 and ARG08, where values exceeded 10,000 ppb, reaching maximum levels close to 15,000 ppb. Additional high concentrations were recorded in ARG19 and ARG20, although at comparatively lower magnitudes.

Oxytetracycline (OTC) also showed notable site-specific peaks. A pronounced concentration spike was observed in ARG10, where levels approached 14,000 ppb. Moderate concentrations were detected in ARG05 and ARG06, whereas most remaining sites showed either low levels or non-detectable concentrations.

Tilmicosin (TIL) was detected at moderate concentrations in selected feedlots, particularly in ARG05 and ARG17, but exhibited a narrower distribution compared to MON and OTC.

Chlortetracycline (CTC) was present in fewer sites and generally at lower concentrations, with localised elevations observed in ARG09 and ARG22. Other antimicrobials—including β-lactams (penicillin –PEN-, ceftiofur –CFT-, cefquinome –CFQ-), fluoroquinolones (enrofloxacin –EFX-, ciprofloxacin -CPX-), macrolides (tulathromycin-TUL-, gamithromycin –GAM-), and sulfonamides (sulfamethoxazole-SMX-, sulfadiazine-SDZ-)—were detected sporadically and predominantly at low concentrations, typically below 2000 ppb, although concentrations were enough for developing bacterial resistance.

**Figure 3:**
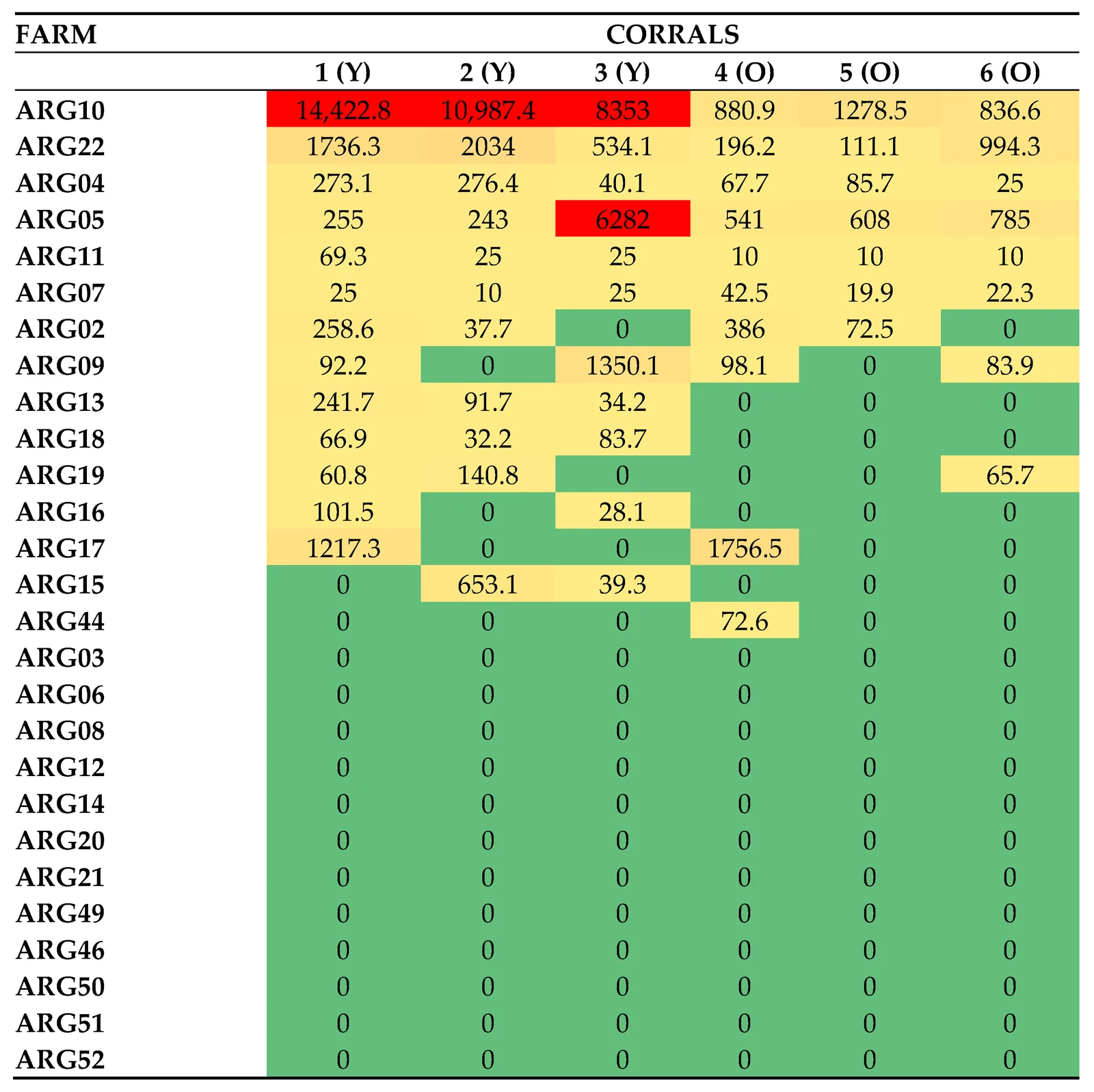
Heatmap of the tetracycline antibiotic concentrations (ppb) in the 27 feedlots (vertical axis) across the three youngest corrals of cattle (1 to 3) and the three oldest corrals (4 to 6) per feedlot in the six columns (horizonal axis). Red color indicates highest concentration, and green color the lowest.

**Figure 4:**
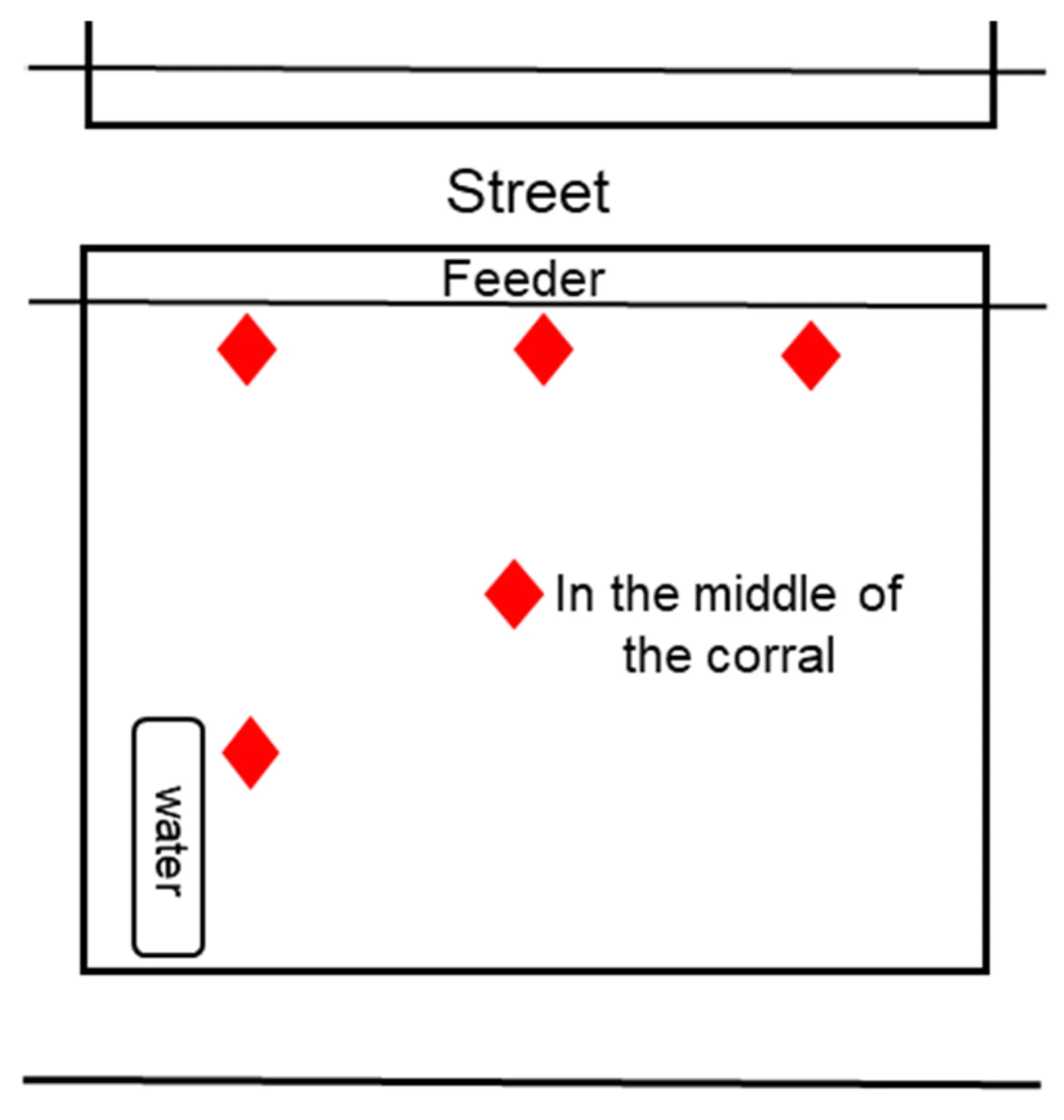
Strategic distribution of soil sampling along the whole trial.

Overall, residue distribution was highly heterogeneous across feedlots, characterised by discrete high-concentration hotspots rather than uniform contamination patterns. The majority of sites exhibited low to moderate concentrations, while a small number of feedlots accounted for the highest residue burdens. MON residue concentrations were not consistently associated with corral type or annual reported level of AMU at the feedlot level. However, tetracycline antibiotic residues were less ubiquitous across the population of feedlots but were exhibited within those feedlots, where they did occur a clear pattern of higher concentrations in the younger age group corrals compared to all other corral types sampled (Figure 3). Among the 27 feedlots, 48.2% had detectable levels of tetracyclines in the youngest cohort of cattle on the farm (corral #1, 2 and 3), falling to 29.6% in the oldest cohort of cattle per farm (corrals #4, 5 and 6) (Figure 4). These also correlate with reported treatment patterns of tetracyclines in new arrivals as part of prophylactic transition protocols. However, the association did not prove to be statistically significant within the mixed effects model. Larger feedlots had a more diverse range of detectable antibiotic residues, where higher annual throughput of cattle had a modest but significantly higher number of antibiotic classes (*p* = 0.02) and R^2^ = 29.87%, and were also associated with a modest but significantly higher total antibiotic residue load (*p* = 0.01) and R^2^ = 38.53%.

### 2.2. Antimicrobial Residues Not Matching Available AMU Records

In 40.7% of feedlots, antimicrobial compounds were not documented in farm-level treatment records; nevertheless, ATM were detected in soil samples across multiple corrals (Table 1). These residues exhibited heterogeneous spatial distribution and variable concentration ranges. This correlated with the biggest farms located in the central region of Argentina (10,000–40,000 heads).

Among the most detected compounds, OTC and TUL showed the highest frequency in 22% of recruited farms. EFX and CPX presented the most pronounced values, reaching concentrations close to 2000 ppb in specific corrals.

β-lactam antibiotics, including AMX and PEN, were identified sporadically across multiple corrals, typically at lower concentrations. Most detections for these compounds were below 200 ppb, although isolated peaks were observed in selected locations. Cephalosporins (third and fourth generation) were also detected at moderate to high levels in several sites, with concentrations generally ranging between LOD and LOQ. The finding of traces of the CFT parent drug as an unreported drug used in four feedlots’ soils is not clearly explained. These concentrations showed in Table 1 were found in sampled feedlots: ARG14 (in three corrals related the longest-residence animals (O1–O3)), in ARG15 (one corral—H-related handling/treatment area), in ARG16 whole sampled corrals and in ARG 17 (1 corral–O1-). However, the likelihood of occasional pouring of pure product in the system (i.e, effluents, manure treatment) might be a potential issue to evaluate.

Macrolides, particularly TUL, exhibited localised high-concentration occurrences, with maximum values approaching 600 ppb in 6/27 feedlots. Their distribution pattern was characterised by discrete hotspots rather than widespread presence.

Sulfonamides, including SFX and SFD, were detected less frequently and generally at low concentrations, commonly between LOD and LOQ.

Overall, the occurrence of these ATMs was highly variable, with most corrals showing either non-detectable levels or low concentrations, while a small subset of sites accounted for the highest residue values.

## 3. Discussion

The present study provides evidence of environmental contamination by veterinary antimicrobials in commercial feedlot soils in Argentina, revealing heterogeneous but measurable residue burdens across production systems. Residue distribution was characterised by marked spatial variability, with discrete high-concentration hotspots rather than uniform contamination patterns (Figure 3). Such heterogeneity has been consistently reported in intensive livestock environments and is attributed to localised manure deposition, treatment practices, and variability in antimicrobial administration across pens [10]. Among detected compounds, MON and OTC exhibited the highest concentrations and widest distribution ranges. Although ionophores such as MON are not classified as critically important antimicrobials for human medicine, their high environmental prevalence reflects intensive use in feedlot production and indicates substantial antimicrobial input into confined animal systems [12,13]. In contrast, tetracyclines remain widely used in both veterinary and human medicine and are known to persist in soil due to strong adsorption to organic matter and limited biodegradation [14]. The elevated concentrations observed in selected sites suggest accumulation processes and environmental persistence consistent with previous findings from manure-impacted soils [7,9]. A particularly notable finding was the detection of multiple antimicrobial residues that were not documented in farm-level AMU records. OTC and TUL were the most frequently detected among these compounds, followed by sulfonamides and several β-lactams (including CFT parent drug) and fluoroquinolones (Table 1). Although the present study was not designed to determine the origin of these discrepancies, several non-exclusive mechanisms may explain their occurrence, including environmental persistence from historical use, manure redistribution between pens, incomplete treatment documentation, extra-label administration, or transfer from adjacent production units. Similar inconsistencies between reported AMU and environmental residue detection have been documented in livestock production systems globally [4]. The environmental presence of critically important antimicrobials for human medicine—particularly fluoroquinolones and advanced cephalosporins—is of special concern within a One Health framework. These antimicrobial classes have high potential for resistance selection, and their detection in soil environments suggests possible selective pressure on environmental microbial communities. In this context, it is important to consider that higher quantification thresholds (LOQ = 200–400 ppb) for some of these critically important antimicrobials (such as advanced cephalosporins and beta-lactams) mean that trace environmental contamination below these levels may be left-censored. Consequently, the reported detection rates for these specific classes represent a conservative estimate of environmental occurrence rather than a definitive absence of residues, potentially underestimating low-level selective pressure in feedlot soils. Numerous studies have demonstrated that even sub-inhibitory concentrations of antibiotics can promote horizontal gene transfer and enrich antimicrobial resistance genes in soil microbiota. Manure-impacted soils are recognised reservoirs and exchange hubs for mobile genetic elements, facilitating the persistence and dissemination of resistance determinants beyond the point of antimicrobial application [7,15]. Although resistome characterisation was beyond the scope of this study, the co-occurrence of multiple antimicrobial classes indicates environmental conditions conducive to resistance selection. From an ecological risk perspective, the magnitude of several detected concentrations suggests that environmental thresholds were exceeded in some localised hotspots. Peak concentrations observed in this study fall within ranges previously associated with microbial community disruption and resistance selection in soils. Furthermore, the simultaneous detection of multiple antimicrobial compounds highlights the importance of considering mixture toxicity, as combined exposure scenarios may result in additive or synergistic ecological effects not captured by single-compound assessments [9,16]. Comparison with international studies indicates that antimicrobial concentrations detected in Argentine feedlot soils are broadly consistent with global observations from intensive livestock production systems. Maximum tetracycline concentrations reported in European and Asian agricultural soils typically range between 1000 and 20,000 ppb, values comparable to the highest levels observed in this study [10,17]. However, the relatively frequent detection of critically important antimicrobials for human medicine in Argentine feedlot soils contrasts with trends reported in some European surveillance programmes, where stricter antimicrobial stewardship policies have reduced environmental exposure [18]. These differences likely reflect variations in antimicrobial usage patterns, regulatory policies, and monitoring systems. The detection of residues not documented in AMU records highlights potential gaps in integrated antimicrobial stewardship and surveillance systems within livestock production. Effective stewardship requires accurate documentation of antimicrobial administration, traceability of treatments, and integration of environmental monitoring into national antimicrobial resistance action plans. Strengthening harmonisation between antimicrobial usage reporting and environmental residue surveillance could improve risk assessment and inform targeted mitigation strategies. In particular, site-specific interventions focusing on high-burden farms and manure management practices may represent efficient approaches to reducing environmental antimicrobial loading.

## 4. Materials and Methods

### 4.1. Study Design and Farm Recruitment

This prospective cross-sectional environmental study established baseline contamination levels and characterised spatial variability across commercial feedlot systems. The farm’s recruitment was undertaken by prioritising the geographic location with the highest density of feedlots, and antibiotic pressure was supposedly higher.

Farms were recruited between 2021 and 2023 via announcements on the Argentine Feedlot Chamber (CAF) website and through veterinarians affiliated with the National Institute of Agricultural Technology (INTA). The survey instrument is described by Bedford et al. (2024) [19].

Antibiotic purchase data were collected by self-reported purchase history for a minimum of the preceding 12 months, annualised and calculated using average animal numbers per farms to give mg/population corrected unit (PCU) using the European Surveillance of Veterinary Antimicrobial Consumption method (ESVAC). Usage of in-feed antibiotics including monensin was calculated from the ration in mg/kg PCU (ESVAC). Twenty-seven farms were selected to carry out the trial based on geographical distribution and large population size (10,000 over 40,000) in Buenos Aires, Cordoba, Santa Fe, and San Luis y Chaco (21 farms), and small-scale feedlots with <1000 cattle, typical of Patagonian production systems (six farms) (See Figure 1).

#### Limitations of the Study

Inherent within the study design is the necessity for feedlot operators to volunteer to participate in the study, which results in a participation bias in the recruitment of a sample population, and thus potentially impacts upon the representivity of the results for the national sector as a whole. However, this limitation is common for observational epidemiological studies globally where good research ethics requires participants to actively agree to participate, and as such reflects good research practice in the field at this point in time. The technical limitations of the laboratory methodologies also inevitably result in limits of detection for each molecule in question; however, these are at the threshold of internationally recognised standards and provide a sound basis for reporting and study. A limited number of sites within each corral could be sampled and a limited number of corrals per farm could be measured due to economic and logistic constraints; however, the sampling protocol provided sufficient datapoints to describe a level of variation that is relevant to clinical, academic and policy makers in their evaluation of the study results.

### 4.2. Sampling

At each feedlot, eight corrals were sampled representing different management categories: one sick-animal pen (S), one handling/treatment area (H), three pens with the most recently arrived animals (Y1–Y3), and three pens with the longest-residence animals (O1–O3). In each corral, five soil subsamples were collected with a clean spoon (Figure 4) and were combined to form a single pooled sample (one pooled sample per corral). Subsamples were placed into 50 mL plastic tubes, homogenised to create the pooled sample, and stored at −20 °C until analysis. Samples were extracted in distinct analytical batches throughout the study period, maintaining processing timeframes within or close to the evaluated stability window (0–90 days). The pooling strategy was chosen to capture corral-level average contamination while limiting analytical costs. Its limitations for detecting fine-scale heterogeneity is acknowledged.

Farm-level AMU data were obtained by standardised questionnaires covering at least the preceding 12 months. Reported purchases were annualised and converted to mg per population correction unit (mg/PCU), following ESVAC methodology. In-feed antibiotic use, including monensin, was estimated from feed rations and expressed as mg/kg PCU.

#### 4.2.1. Antimicrobial Residue Quantification in Soil Samples

Soil samples were analysed to quantify amoxicillin (AMX), penicillin (PEN), ampicillin (AMP), ceftiofur (CTF), cefquinome (CFQ), sulfamethoxazole (SMX), enrofloxacin (EFX), ciprofloxacin (CPX), tulathromycin (TUL), gamithromycin (GAM), tilmicosin (TIL), sulfadiazine (SDZ), oxytetracycline (OTC), chlortetracycline (CTC), and monensin (MON) using Ultra-Fast Liquid Chromatography (UFLC) coupled with tandem mass spectrometry (MS/MS), following a methodology developed in our laboratory. This protocol represents a specific methodological approach tailored to the simultaneous analysis of the antimicrobial compounds investigated in the present study.

#### 4.2.2. Chemicals and Reagents

Antimicrobial reference standards (99% purity) were sourced from Sigma-Aldrich and prepared as 1,000,000 ppb methanolic stock solutions. HPLC-grade solvents and reagents were obtained from Sigma-Aldrich: Trichloroacetic acid (TCA) and ethylenediaminetetraacetic acid (EDTA); Baker: Acetonitrile (ACN), methanol (MeOH), and ammonium formate; Merck: (hexane); Biopack: formic acid, and Agilent Technologies: Primary Secondary Amine (PSA). Working standards (10–100,000 ppb) were prepared weekly, and purified water was obtained via a Millipore Simplicity system.

#### 4.2.3. Soil Sample Extraction

For AMX, PEN, AMP, CEF, CFQ, SMX, EFX, TUL, GAM, CFX, and SDZ residues, an aliquot (0.1 g) of soil sample was extracted by adding 100 µL of water containing 2% formic acid. After stirring briefly, 150 µL of 0.1 M EDTA and 1000 µL of acetonitrile (ACN) with 2% formic acid were added. The mixture was then vortexed at high speed for 10 min and sonicated for 30 min. Finally, samples were centrifuged at 15,000 rpm for 10 min at 1 °C. The clear supernatant was filtered through a 0.22 µm nylon filter, and 10 µL of the filtrate was injected into the chromatographic system. The extraction of OTC and CTC residues involved shaking a 0.4 g aliquot for 45 min after adding 250 μL of 0.1 M EDTA and 1000 μL of a 5% TCA/ACN solution (*v*/*v*, 1:1). Following sonication (45 min) and ultracentrifugation (15,000 rpm, 10 min), a 1600 μL supernatant aliquot was combined with 1000 μL of hexane and 0.05 g of PSA. After gentle shaking for 10 min and centrifugation for 10 min at 0 °C, an aliquot of the aqueous phase was taken. The sample was then ultracentrifuged for 15 min at 15,000 rpm, and a 1 mL aliquot was filtered through a 0.22 μm nylon membrane. A 10 μL portion was injected into the chromatographic system.

TIL and MON residues were extracted by adding 250 µL of water to a 0.1 g analytical portion. After brief mixing, 1 mL of cold ACN was introduced. The mixture was shaken for 45 min, sonicated for 30 min, and then ultracentrifuged at 15,000 rpm for 10 min at 4 °C. The resulting supernatant was transferred to 5 mL glass tubes and evaporated to dryness using a nitrogen stream at 56 °C. The dried sample was then resuspended in 1 mL of ACN:5 mM ammonium formate (60:40). After vortexing and centrifugation, the sample was filtered through a 0.22 μm nylon membrane, and a 10 μL aliquot was injected into the chromatographic system.

#### 4.2.4. UFLC-MS/MS System and Chromatographic Conditions

Analysis was performed using a Shimadzu UFLC system coupled to an LCMS-8050 triple quadrupole mass spectrometer. Separation was achieved on a Shim-pack HR-ODS C18 column (15 cm × 3 mm, 2.6 μm) at 40 °C. The ESI source operated with interface, desolvation, and heat-block temperatures of 300 °C, 250 °C, and 400 °C, respectively. MRM transitions (quantifier and qualifier) and collision energies were optimised via direct injections (0.1 mg/mL) for each compound. Distinct gradient programmes were used for each analyte group. For most residues (AMX through SDZ), a water/ACN (0.1% formic acid) phase at 0.2 mL/min was used, starting at 50% B and reaching 80% B by 3 min. For OTC and CTC, the flow rate was 0.4 mL/min with a 10% to 50% ACN gradient over 5 min. Finally, TIL and MON were separated using 5 mM ammonium formate and ACN (0.4 mL/min) with a 60% to 95% B gradient over 14 min.

#### 4.2.5. Methodology Validation

The analytical method was validated for all 15 antimicrobials in soil, assessing linearity, recovery, precision, accuracy, stability, limit of quantification (LOQ), and matrix effects. Blank soil samples for the method validation were taken in a separate farm from 3 corrals with records of free animals. Matrix-matched calibration curves showed high linearity (r ≥ 0.985) (see Table 1). Mean absolute recoveries ranged from 30% to 95%. The variability in recovery among compounds was considered in relation to the multiresidue nature of the method, as the extraction procedure was applied to compounds with different physicochemical properties and, consequently, different extraction efficiencies. The recovery results were evaluated together with the other validation parameters to assess the overall performance of the method (Table 2). Both intra-day and inter-day precision and accuracy met acceptable criteria (CV and RE within −40% to +20% for low levels; −20% to +10% for levels ≥ 100 ppb). Matrix effects were negligible (80–120%), and the limit of determination (LOD) and LOQs obtained for each analyte is shown in Table 1. The LOD enabled scientific verification of whether the antibiotic was absent or remained below the instrument’s detection limit in specific samples. High-performance analytical sensitivity (LOQ = 1–25 ppb) was achieved for ubiquitous compounds such as MON, TIL, and tetracyclines (OTC/CTC), enabling precise trace detection and robust distribution profiling even at ultra-low environmental concentrations. Conversely, higher LOQs (50–400 ppb) observed for other compounds, particularly beta-lactams, advanced cephalosporins, and certain macrolides, were driven by matrix suppression and extraction challenges inherent to organic soil. The long-term stability of each compound was evaluated using blank soil samples fortified at two concentration levels of each compound and stored at −20 °C. Samples (*n* = 3) were analysed at 0 and 90 days post-freezing. Stability, expressed as %CV, remained within the expected parameters (±15%).

An analytical LOD sensitive enough to quantify sub-inhibitory antimicrobial concentrations is crucial, as even trace levels can exert selective pressure that promotes the emergence and dissemination of antimicrobial resistance genes. For this reason, this parameter was considered pivotal for determining soil ATM contamination [5].

#### 4.2.6. Statistical Analysis

Data were analysed in Minitab Statistical Software 22 Minitab. Descriptive analysis was performed first, followed by univariate comparison of residue concentrations of each of the antibiotic classes against corral type using the Kruskal–Wallis Test to account for the nonparametric nature of the data distributions and linear regression to assess correlations between feedlot size and antibiotic residue class diversity, followed by mixed effects modelling of residue concentration of each antibiotic class as the independent variable with farm corral type within farms as dependent variables, along with the annual cattle population of the farm and the antibiotic usage of the farm. Antibiotic concentration distributions were normalised by an optimised Box–Cox transformation to the power (−0.16). Model fit was checked graphically.

## 5. Conclusions

This study documents the presence and spatial heterogeneity of veterinary antimicrobial residues in Argentine feedlot soils, including compounds of clinical importance. The findings identify discrete hotspots and highlight the need for improved AMU recordkeeping, targeted environmental monitoring, and interventions to reduce environmental loading. These data provide a baseline for future resistome analyses and One Health policy development.

## Figures and Tables

**Table 1:** Average of concentrations (ppb) of ATM compounds not documented in farm-level treatment records found in corral soils. The distribution of combined ATM molecules found in 11/27 recruited farms were: six different unreported ATMs in one farm, five different unreported ATMs in one farm, four different unreported ATMs in four farms, three different unreported ATMs in two farms, two different unreported ATMs in two farms, and one different unreported ATM in one farm.

ATM ^1^	Farms Recruited	Farms with Antimicrobial Residues Not Matching Available AMU Records	Residues Detected in Soil (ppb) Mean (Range)
OTC	27	6 (22%)	643.5 (35–1217)
CTC	27	1 (3%)	26.5 (0)
AMX	27	4 (14%)	≤LOQ
PEN	27	4 (14%)	=LOD
CFT	27	4 (14%)	≤LOQ
CFQ	27	3 (11%)	=LOD
SMX	27	4 (14%)	=LOD
SDZ	27	1 (3%)	≤LOQ
EFX	27	2 (7%)	596.5 (134–925)
CPX	27	2 (7%)	1448 (369–2158)
TUL	27	6 (22%)	501 (400–602)
GAM	27	4 (14%)	<LOD

^1^ Amoxicillin (AMX), penicillin (PEN), ceftiofur (CTF), cefquinome (CFQ), sulfamethoxazole (SMX), sulfadiazine (SDZ), enrofloxacin (EFX), ciprofloxacin (CPX), tulathromycin (TUL), gamithromycin (GAM), oxytetracycline (OTC), chlortetracycline (CTC).

**Table 2:** Individual antimicrobials’ main validation parameters obtained in the multiresidue method for feedlot soils samples: Limit of Detection (LOD), Limit of Quantification (LOQ), Correlation Coefficient (R^2^) and Recovery (R).

ATM	LOD (ppb)	LOQ (ppb)	R^2^	R (%)	Intra-Day Precision (RSD,%)
MON	10	25	0.9995	79	4.437
OTC	0.5	1	0.9996	65	10.788
CTC	0.5	1	0.9986	79	15.126
AMX	200	400	0.9874	95	7.076
PEN	25	50	0.9932	91	5.646
AMP	100	200	0.9821	88	3.112
CFT	25	50	0.9889	85	12.677
CFQ	200	400	0.9977	72	16.258
SMX	50	100	0.9985	68	6.264
SDZ	25	50	0.9951	75	13.277
EFX	25	50	0.9984	40	15.047
CPX	50	100	0.9903	30	16.810
TUL	200	400	0.9990	35	12.268
GAM	50	100	0.9978	35	16.098
TIL	10	25	0.9976	89	13.124

Amoxicillin (AMX), penicillin (PEN), ampicillin (AMP), ceftiofur (CTF), cefquinome (CFQ), sulfamethoxazole (SMX), sulfadiazine (SDZ), enrofloxacin (EFX), ciprofloxacin (CPX), tulathromycin (TUL), gamithromycin (GAM), tilmicosin (TIL), oxytetracycline (OTC), chlortetracycline (CTC), and monensin (MON). R^2^ is the coefficient of determination for matrix-matched calibration.

## Data Availability

The data that support the findings of this study are available from the corresponding author upon reasonable request.

## Abbreviations

The following abbreviations are used in this manuscript:

ATM	Antimicrobials
AMR	Antimicrobial resistance
UFLC-MS/MS	Ultra-fast liquid chromatography coupled with tandem mass spectrometry
AMU	Antimicrobial use
PCU	Population corrected unit
MON	Monensin
OTC	Oxytetracycline
TIL	Tilmicosin
PEN	Penicillin
AMP	Ampicillin
CFT	Ceftioufur
CFQ	Cefquinome
EFX	Enrofloxacin
CPX	Ciprofloxacin
TUL	Tulathromycin
GAM	Gamithromycin
SMX	Sulfamethoxazole
SDZ	Sulfadiazine
TCA	Trichloroacetic acid
EDTA	Ethylenediaminetetraacetic acid
ACN	Acetonitrile
MeOH	Methanol
PSA	Primary secondary amine
LOQ	Limit of quantification
LOD	Limit of determination
